# Computer Vision Approach for the Determination of Microbial Concentration and Growth Kinetics Using a Low Cost Sensor System

**DOI:** 10.3390/s19245367

**Published:** 2019-12-05

**Authors:** Marco Grossi, Carola Parolin, Beatrice Vitali, Bruno Riccò

**Affiliations:** 1 Department of Electrical Energy and Information Engineering “Guglielmo Marconi” (DEI), University of Bologna, 40136 Bologna, Italy; bruno.ricco@unibo.it; 2 Department of Pharmacy and Biotechnology (FaBiT), University of Bologna, 40127 Bologna, Italy; carola.parolin@unibo.it (C.P.); b.vitali@unibo.it (B.V.)

**Keywords:** sensor, bacteria, computer vision, portable system, plate count

## Abstract

The measurement of microbial contamination is of primary importance in different fields, from environmental monitoring to food safety and clinical analysis. Today, almost all microbiology laboratories make microbial concentration measurements using the standard Plate Count Technique (PCT), a manual method that must be performed by trained personnel. Since manual PCT analysis can result in eye fatigue and errors, in particular when hundreds of samples are processed every day, automatic colony counters have been built and are commercially available. While quick and reliable, these instruments are generally expensive, thus, portable colony counters based on smartphones have been developed and are of low cost but also not accurate as the commercial benchtop instruments. In this paper, a novel computer vision sensor system is presented that can measure the microbial concentration of a sample under test and also estimate the microbial growth kinetics by monitoring the colonies grown on a Petri dish at regular time intervals. The proposed method has been in-house validated by performing PCT analysis in parallel under the same conditions and using these results as a reference. All the measurements have been carried out in a laboratory using benchtop instruments, however, such a system can also be realized as an embedded sensor system to be deployed for microbial analysis outside a laboratory environment.

## 1. Introduction

Microbial concentration detection is of primary importance in many fields of application such as environmental monitoring [1,2], food safety [3,4] and clinical analysis [5,6]. The presence of high microbial contamination, or even the presence of small concentrations of highly dangerous pathogens (such as *Escherichia coli O157:H7* and *Salmonella typhi*) can represent a serious threat to human health. In this regard, approximately 48 million infections have been reported in a single year in the United States of America by the Center for Disease Control and Prevention (CDC) [7].

The standard technique to measure microbial concentration is the Plate Count technique (PCT) [8]. The sample to be analyzed is diluted in different ratios and inoculated in sterile Petri dishes filled with a nutrient agarised medium to allow microbial replication. After an incubation of 24–72 h, the number of colonies grown on the Petri dish are counted and the microbial concentration estimated as colony forming units per milliliter (CFU/mL). PCT is essentially a laboratory method that is accurate but also time consuming and difficult to be implemented outside a laboratory.

Much research has been carried out in recent years to provide low-cost and quick alternatives for microbial concentration detection. Techniques such as quantitative Polymerase Chain Reaction (qPCR) [9,10] and Matrix-Assisted Laser Desorption Ionization Time-Of-Flight (MALDI-TOF) mass spectrometry [11,12] can provide a near real-time response and can also detect non-culturable cells. However, these techniques must be carried out by trained personnel and require expensive instrumentation. Biosensors, on the other hand, can provide good selectivity towards particular microbial strains with quick response time (<1 h); in a biosensor, a biorecognition element (bioreceptor) is integrated on the sensor to allow the target cell to bind to the bioreceptor [13,14] and the cell binding can be detected using different transduction principles, such as impedance-based [15], potentiometric [16] and amperometric [17]. Biosensors’ main drawbacks are the short lifetime (because of low stability of the biorecognition element) and complex procedures for the bioreceptor immobilization. Other techniques have been also proposed which (although the response time is not as short as biosensors) are easier to be implemented in an automatic form. One such example is Impedance Microbiology (IM), a technique based on Electrical Impedance Spectroscopy (EIS) [18], where the microbial concentration is estimated from the time needed for a microbial population to reach a threshold concentration. Microbial metabolism produces changes in the culture medium electrical properties and such changes are measured to detect when the microbial population has reached the threshold concentration. IM can measure the microbial concentration with response times in the range from 3 to 14 h (depending on the particular strain and initial microbial concentration) that are shorter than PCT (24–72 h). IM has been successfully implemented to detect microbial concentration in different types of products, including dairy-based desserts [19], raw milk [20], vegetables [21], meat [22], water [23], coolant and lubricant products for machining processes [24], and to investigate the inhibition efficiency of antibiotics and biocides on bacterial growth [25]. An innovative microfluidic implementation of IM was proposed in 2005 concentrating microbial cells using dielectrophoresis, thus strongly reducing the response time [26]. More recently, a combined approach of IM and optical turbidity measurement has been proposed to measure the microbial concentration that, in some cases, can also discriminate the different types of microorganisms present in the analyzed sample [27].

Despite the many innovative techniques proposed to measure microbial concentration, PCT is still the reference method commonly used in microbiology laboratories, where tens or even hundreds of Petri dishes are processed every day for microbial analysis. PCT is normally used in laboratories where colony count is manually carried out by qualified personnel. When many plates are processed, this can lead to eye fatigue and produce errors. Automatic colony counters have been proposed in the literature [28,29], are also commercially available [30,31] and can perform the counting process in an automatic way, thus drastically reducing the analysis time and the chance of error. Since these systems are usually expensive, recently different apps have been designed to turn smartphones into an automatic colony counter by exploiting the camera embedded in modern mobile phones [32,33,34]. While smartphone-based colony counter apps are usually very cheap (or even available for free) and their accuracy is usually much lower than commercially available colony counter systems.

In this paper, a computer vision system is presented; it can measure the microbial concentration and also estimate the growth kinetics. The technique is based on PCT, where an inoculated Petri dish is incubated and monitored with a low-cost camera to detect the colonies during the growth phase, thus counting their number and estimating colony size. The proposed method is implemented automatically using a laptop PC that controls the camera by USB communication.

The paper is organized as follows. In Section 2, the computer vision system is presented, and the microbiological analysis used to estimate its accuracy is discussed. In Section 3, the results of measurements with the system are presented and discussed. Finally, conclusions are drawn in Section 4.

## 2. Materials and Methods

A computer vision sensor system was developed to estimate the microbial concentration as well as the microbial growth kinetic parameters. The system was tested with different microbial species (*Escherichia coli*, *Staphylococcus aureus*, *Pseudomonas aeruginosa*, *Saccharomyces cerevisiae*) cultured in different growth media. The designed system was in-house validated by carrying out PCT assays in parallel under the same conditions.

### 2.1. The Computer Vision Sensor System

**Figure 1:**
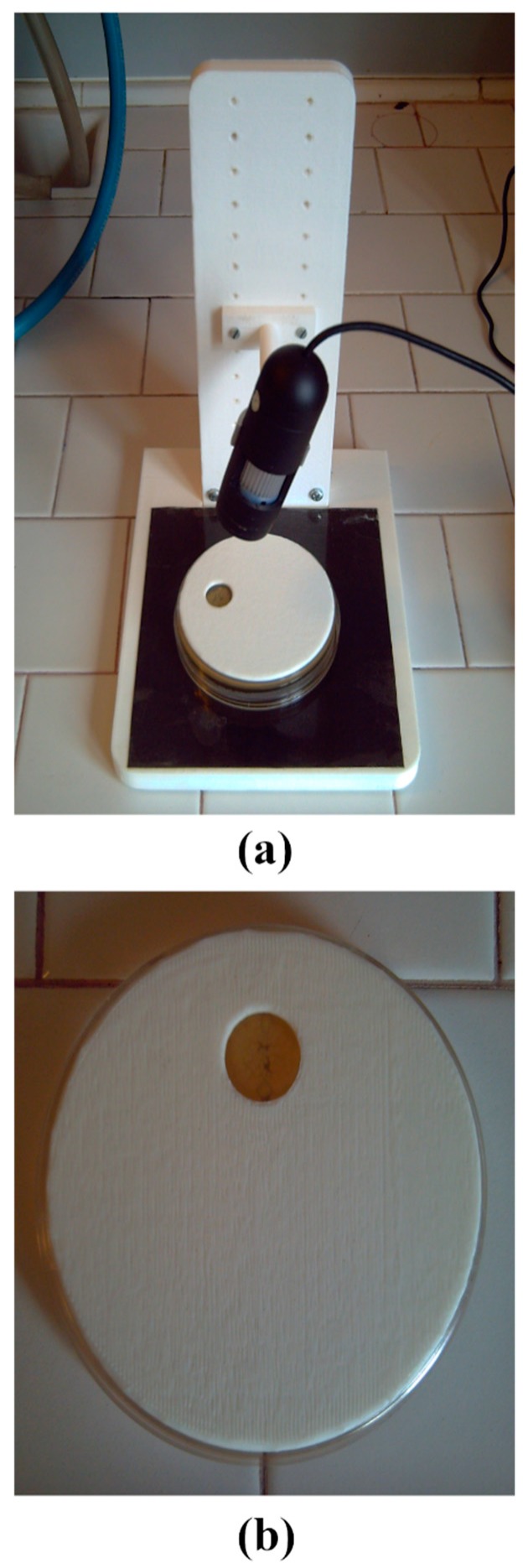
Picture of the proposed computer vision sensor system for microbial analysis (**a**) and designed mask to select the area of the inoculum (**b**).

The computer vision sensor system was built using a low-cost camera (Proxima USB 2.0). A picture of the system is presented in Figure 1a. The camera is held in position using an ad-hoc designed structure (realized with a 3D printer, Makerbot Z18 by Makerware) capable to set the distance to the Petri dish at different values. In each assay the Petri dish was inoculated with 20 µL of the sample under test. The inoculum was placed in a small circular area (15 mm diameter) using an ad-hoc designed mask, created with a 3D printer as shown in Figure 1b. The camera was programmed to acquire images of the Petri dish at time intervals of 10 min with a resolution of 640 × 480. The camera magnifying ratio was set to 32.7 µm/pixel.

The low-cost camera was placed in a thermal incubator (Binder) set to the target temperature and controlled by a USB port using a laptop PC. The software to control the camera for both acquisition and analysis was written using the Processing programming language. Each assay was carried out with the following steps: the Petri dish was inoculated and placed under the camera inside the thermal incubator; a photo of the inoculated area (circular area of 15 mm diameter as shown in Figure 1b) was taken every 10 min for a total of 24–48 h (depending on the analysed microbial species) and saved on the PC hard disk; at the end of the assay, the images were processed to extract the information parameters (number of colonies and total colonies area).

The acquired images were processed according to the following algorithm:

− the image is blurred using a blur parameter of 5,
− the image is converted from RGB to grayscale,
− an image is generated as the difference of the current image and the image acquired at time 0,
− the new generated image is binarized using a threshold of 0.07,
− the number of colonies and the total colonies area for the binarized image are determined.

The colonies in the binarized image were detected using a blob detection algorithm whose code is presented in [35]. Preliminary measurements were carried out to compare the results achieved using the blob detection algorithm with those obtained with the circle Hough transform algorithm [36] implemented using the library OPENCV in Python. Although the Hough transform algorithm is capable to discriminate two overlapping colonies while the blob detection algorithm detects them as a single blob, the blob detection algorithm resulted in more accurate detection and higher correlation with the reference PCT measurements, at least in the beginning of the colonies growth phase, when the colonies are perfectly separated. This can be explained by the fact that some colonies are not perfectly circular and their transparency level is different for different microbial species and growth media. Thus, in the following, all images were processed using the blob detection algorithm.

**Figure 2:**
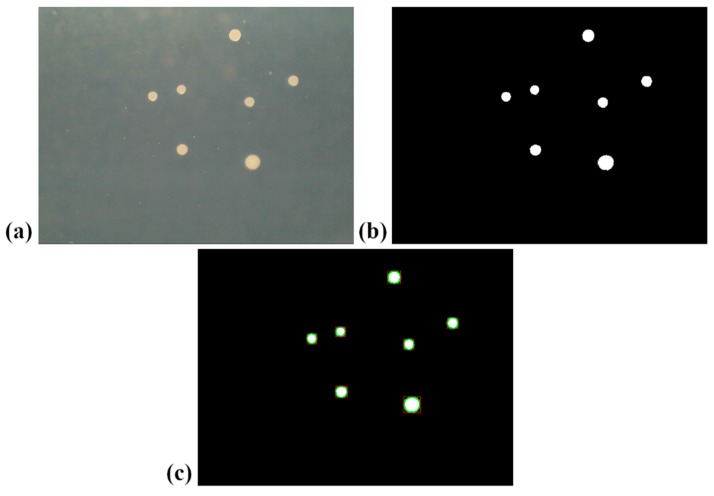
Pictures of the colonies grown on the Petri dish in the case of the microbial specie *Saccharomyces cerevisiae*. Picture taken by the camera (**a**), after the binarization process (**b**) and after the application of the blob detection algorithm (**c**).

In Figure 2, the different phases of the image processing are shown in the case of a sample contaminated with 350 CFU/mL of *S. cerevisiae*: the original acquired image is presented in Figure 2a, and the binarized image in Figure 2b, while the binarized image with the colonies detected in Figure 2c. The colony area was determined by the number of white pixels inside the detected blob. The total colonies area was calculated as the sum of the colony area for all detected colonies. To avoid erroneous detection of colonies due to light reflection effects and scratches on the Petri dish, all detected blobs featuring an area lower than 40 pixels were discarded.

### 2.2. Microbiological Analysis

Four different laboratory cultured microbial species were used for the tests carried out using the computer vision system presented in this work: *Escherichia coli* ATCC 11105, *Staphylococcus aureus* ATCC 29213, *Pseudomonas aeruginosa* DSM 50071, *Saccharomyces cerevisiae* ATCC 26785. *E. coli*, *S. aureus*, *P. aeruginosa* were routinely cultured in Nutrient broth at 37 °C, with vigorous shaking; *S. cerevisiae* was routinely cultured in Sabouraud Dextrose broth at 30 °C, with vigorous shaking. Overnight cultures of each microorganism were prepared, serially diluted in sterile saline (0.9% NaCl) and inoculated on Petri dishes filled with agarised appropriate growth medium (medium added with 1.5% agar), and placed under the camera. *E. coli* and *S. aureus* dilutions were spotted (20 µL) on Nutrient (NA) or Brain Hearth Infusion (BHI) agar plates; *P. aeruginosa* dilutions were spotted on NA plates, plates were incubated at 37 °C for 24 h. *S. cerevisiae* dilutions were spotted on Sabouraud Dextrose agar (SDA) plates and incubated at 30 °C for 48 h.

For each tested sample, the microbial concentration was also measured using the standard PCT under the same conditions (growth medium and temperature) and the result used as reference to evaluate the accuracy of the proposed computer vision sensor system.

All media components were from Becton, Dickinson and Company (Sparks, MD, USA).

## 3. Results and Discussion

The sensor system has been tested with different laboratory cultured microbial strains and the results compared with PCT analysis carried out in parallel for reference. A total of 23 samples have been tested (each featuring different microbial concentrations): three samples for *E. coli* in NA, nine samples for *E. coli* in BHI, three samples for *S. aureus* in NA, two samples for *S. aureus* in BHI, two samples for *P. aeruginosa* in NA, four samples for *S. cerevisiae* in SDA. Each sample was tested in duplicate and the results averaged. In each assay the number of colonies grown on the Petri dish and the growth kinetics of such colonies are measured.

### 3.1. Determination of the Algorithm Parameters

As discussed in Section 2.1, each acquired image is processed using a computer vision algorithm composed of different steps, including a blur transformation and image binarization using a threshold. The optimal values for the parameters of such transformations have been determined by testing different values and comparing the achieved accuracy.

**Figure 3:**
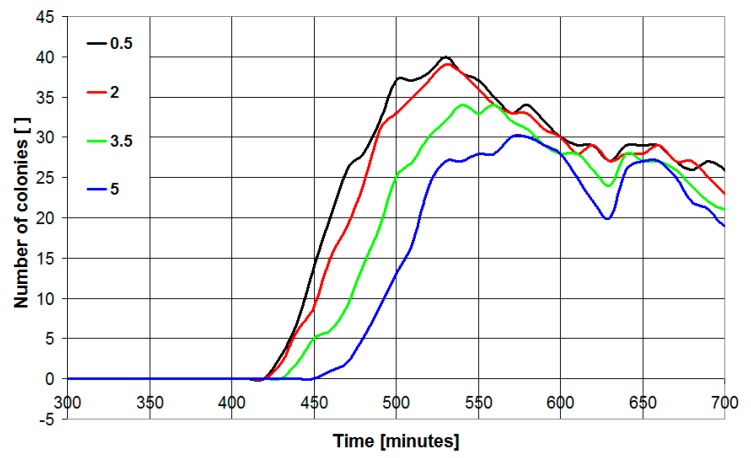
Number of detected colonies as a function of time in the case of *Escherichia coli* plated on BHI medium (microbial concentration 2500 CFU/mL). The algorithm has been implemented with a binarization threshold parameter of 0.07 for different values of the blur parameter (0.5–5).

In the case of blurring, the transformation provides better results since it helps to remove image artefacts (such as scratches and light reflections on the Petri dish) that can result in erroneous colony detection. However, very high values of the blur parameter can result in underestimation of the number of colonies. This is shown in Figure 3, where four different blur parameters (0.5, 2, 3.5 and 5) have been tested in the case of a sample contaminated with *E. coli* and tested in BHI medium using a binarization threshold of 0.07. As can be seen, the number of detected colonies decreases with the increase of blur parameter. It is 40, 39, 34 and 30 for a blur parameter of 0.5, 2, 3.5 and 5, respectively. The blur parameter has negligible effects on the measured covered area. Thus, a value of 0.5 for the blur parameter has been used.

**Figure 4:**
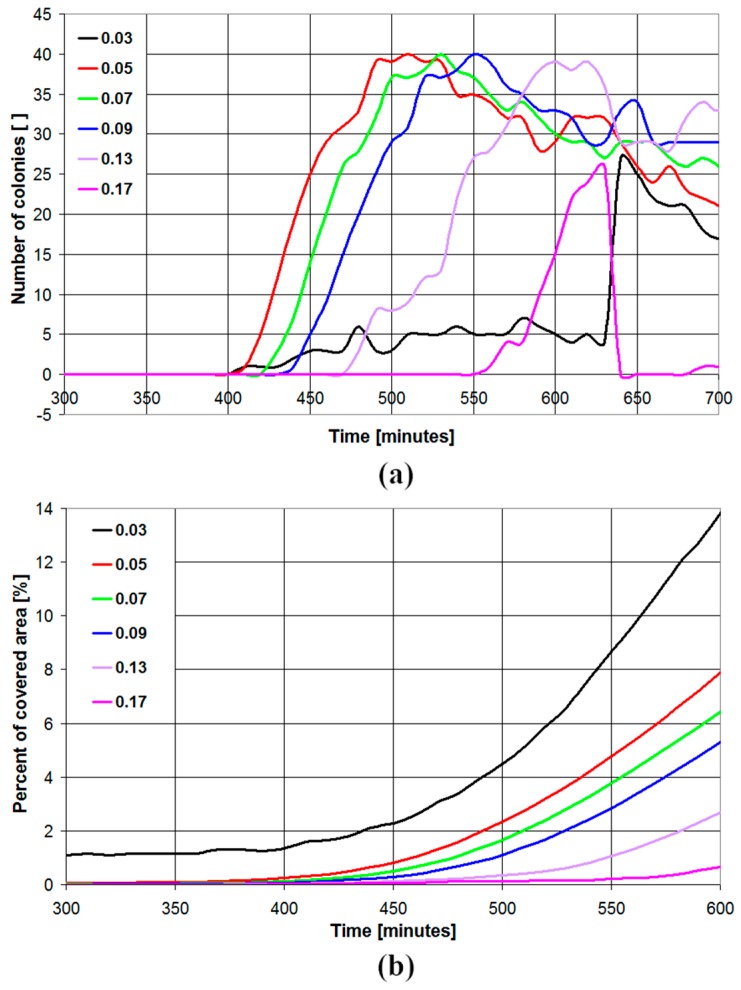
Number of detected colonies (**a**) and percent of covered area (**b**) as a function of time in the case of *Escherichia coli* plated on BHI medium (microbial concentration 2500 CFU/mL). The algorithm has been implemented with blur parameter of 0.5 for different values of the binarization threshold parameter (0.03–0.17).

The binarization threshold parameter is of paramount importance to achieve high accuracy in colony detection. In fact, different microbial strains and different growth media result in different levels of transparency for the colonies grown on the Petri dish. In Figure 4, the number of detected colonies and the percent of covered area are plotted vs. time in the case of pictures processed with a blur parameter of 0.5 and different values for the binarization threshold parameter (0.03, 0.05, 0.07, 0.09, 0.13, 0.17) for a sample contaminated with *E. coli* and tested in BHI medium. As can be seen, very low values of the binarization threshold parameter (0.03) result in poor detection of the number of colonies and a large offset at time 0 for the percent of covered area since many background pixels are erroneously interpreted as colony pixels. On the other hand, high values of the binarization threshold parameter (0.17) also result in poor detection of the number of colonies, since many colonies pixels are erroneously interpreted as background pixels. Moreover, increasing the value of the binarization threshold parameter, the number of detected colonies reaches the maximum value at higher times, thus resulting in longer detection times. The optimal binarization threshold parameter was set to 0.07, based on investigations on different microbial species and growth media.

### 3.2. Evaluation of the Algorithm Accuracy

The computer vision algorithm discussed in Section 2.1 has been used to evaluate the microbial concentration and growth kinetics for the microbial species presented in Section 2.2. For all microbial species, different microbial concentrations have been tested and the results compared with PCT measurements were carried out in parallel under the same conditions.

**Figure 5:**
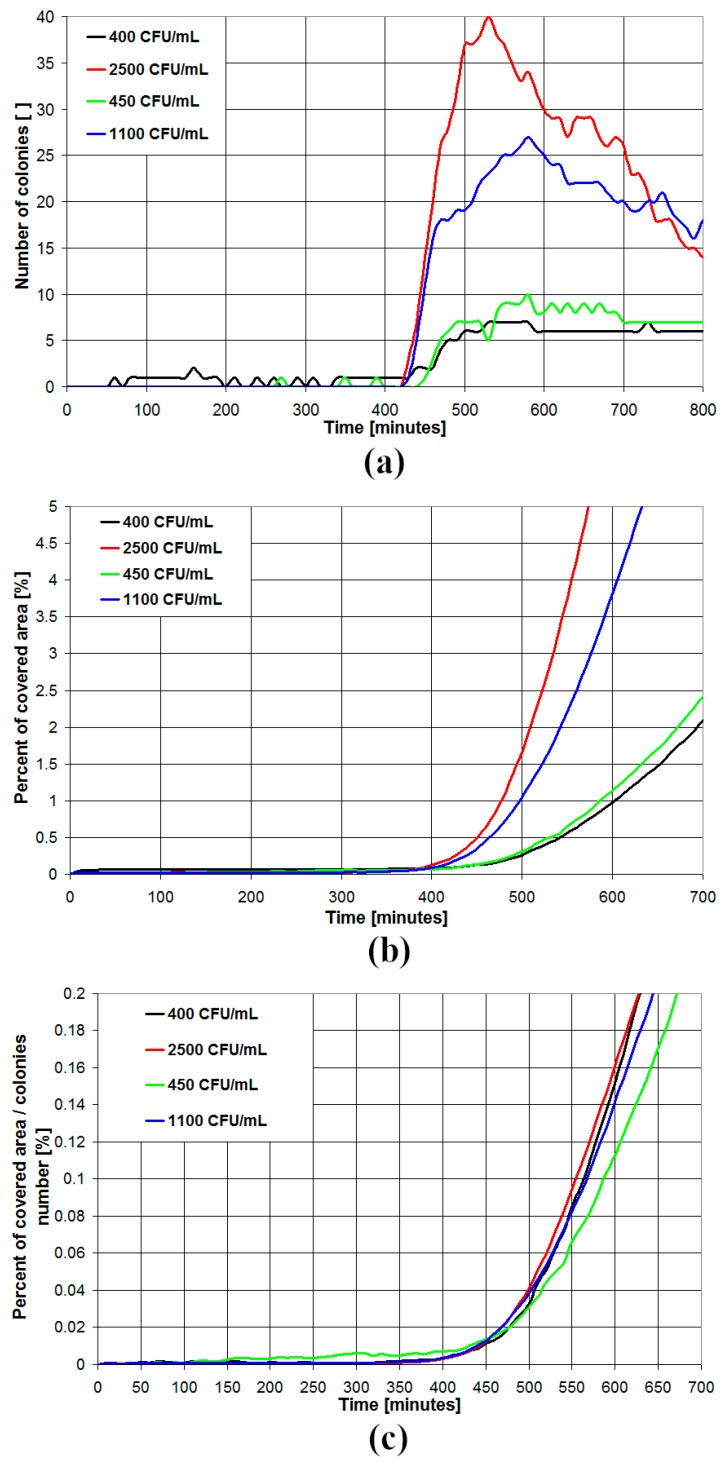
Number of detected colonies (**a**), percent of covered area (**b**) and percent of covered area for a single colony (**c**) as a function of time in the case of *Escherichia coli* plated on BHI medium for different microbial concentrations of the inoculum.

In Figure 5, the results for different concentrations of *E. coli* in BHI medium are presented. As can be seen in Figure 5a, the number of detected colonies is initially zero, then increases with time, reaching a maximum and then decreases over time. This is the result of the growth of colonies on the Petri dish. Initially, the colonies are too small to be detected, then their size increases and the number of detected colonies increases too. However, when the colony size becomes too big, the colonies overlap each other, and the algorithm detects them as a single colony. Thus, the number of detected colonies starts to decrease. The number of colonies for the tested sample is estimated from the maximum number of detected colonies during the assay. As expected, samples featuring higher values of the microbial concentration are also characterized by higher values of the maximum number of detected colonies. The percent of covered area (Figure 5b) represents the percent of screen area that is covered by colonies. As expected, this area is initially zero and then increases over time, at least until the colony size stops to increase due to the lack of nutrients. The percent of covered area starts to increase at a time that is characteristic of the particular microbial species but its rate of increase is also a function of the number of colonies and thus of the sample microbial concentration. However, if the percent of covered area is divided by the number of detected colonies (Figure 5c), this parameter is not dependent on the sample microbial concentration and is characteristic only of the particular microbial species.

The percent of covered area for a single colony has been fitted using a piecewise linear function *f*(*t*) defined as:

(1) $$f\left(t\right)=\{\begin{array}{c} a\\ a+c\left(t-b\right) \end{array}\;\begin{array}{c} t\leq b\\ t>b \end{array}$$

In the fitting function f(t), the parameter a accounts for an offset due to artefacts in the acquired images and has no correlation with the microbial kinetics growth, parameter b accounts for the lag time needed for the colonies to reach a size to be detected, while parameter c accounts for the growth speed of the colonies. The fitting function of Equation (1) has been used for all tested microbial species in the time range when no colonies overlapping occours (i.e., percent of covered area for a single colony < 0.2%). In all cases, the achieved coefficient of determination R^2^ was higher than 0.98, indicating a very good fit for the measured data.

**Figure 6:**
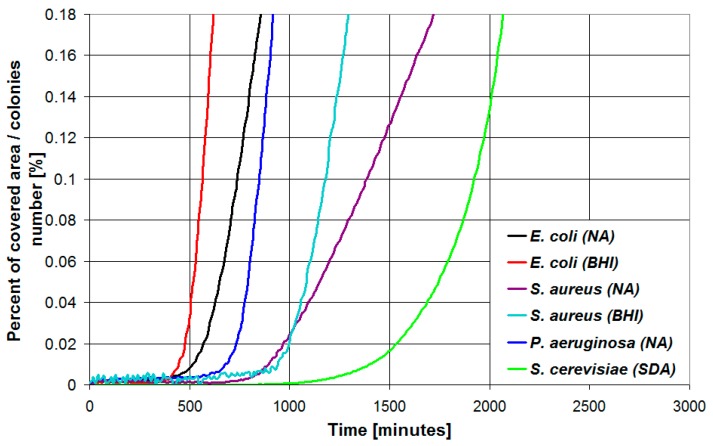
Percent of covered area for a single colony plotted vs. time in the case of the different microbial species and growth media investigated in this work.

The percent of covered area for a single colony is plotted in Figure 6 for the different microbial species and growth media investigated in this work and the corresponding parameters of the fitting function of Equation (1), estimated using the software CurveExpert Professional 2.4, are presented in Table 1. As can be seen, different microbial species feature different values for the lag time parameter and growth speed parameter: *E. coli* in BHI is the fastest growing microbial species among the ones tested, while *S. cerevisiae* features a low growth rate and also the longest lag time. Considering the same microbial species, different growth media are characterized by different values of the kinetics parameters. For example, in the case of *E. coli*, BHI medium provides higher growth rate and shorter lag time if compared to NA medium. In the case of *S. aureus* the growth rate in BHI medium is more than twice the growth rate in NA medium, while both media provide comparable values for the lag time.

The estimation of microbial species growth rate and lag time is very important in different field of applications, for example when testing the efficiency of microbial biocides to evaluate how different concentrations of the compound affect the ability of the cells to grow, or when the optimal growth temperature must be determined for a particular microbial species/growth medium combination.

**Figure 7:**
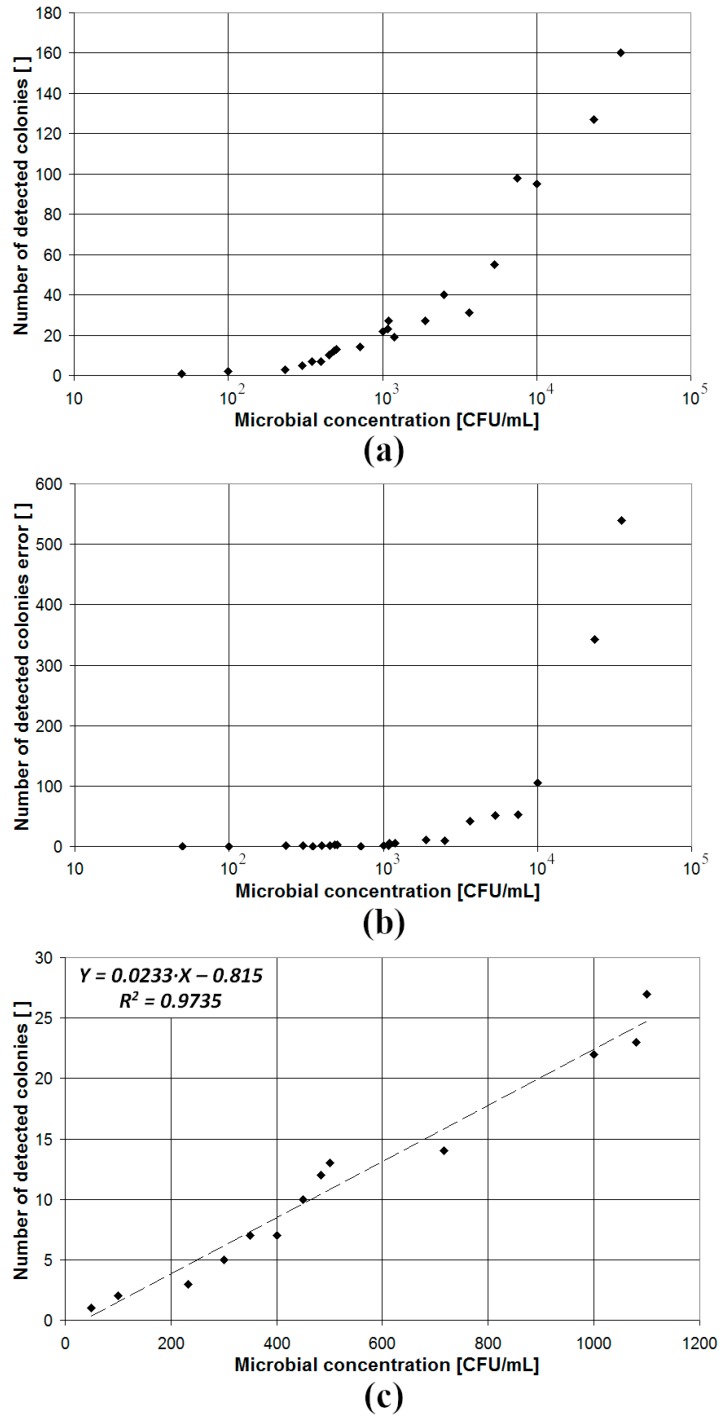
Scatter plot representing the number of detected colonies (**a**) and the error of the number of detected colonies (**b**) vs. the microbial concentration measured by the reference PCT assay in the case of the full set of investigated samples and the number of detected colonies vs. the microbial concentration for a subset of samples featuring a microbial concentration lower than 1200 CFU/mL (**c**).

The results of the accuracy of the proposed system in the determination of the microbial concentration are presented in Figure 7a, where the number of colonies detected by the proposed system are plotted vs. the microbial concentration determined by a PCT assay carried out in parallel. The data are reported for all the assays (different microbial species and growth media) tested in this work. Each dot of the scatter plot represents the outcome of a single assay (number of colonies detected by the proposed system on the *y*-axis and microbial concentration determined by a PCT on the *x*-axis) without any discrimination for different microbial species and/or growth media. As expected, the number of detected colonies increases with the microbial concentration measured by PCT. However, as the sample microbial concentration increases, the error in the number of detected colonies also increases, since when the number of colonies is high, there is a high probability of colonies overlapping in the early phase (when the colonies are still small), thus resulting in underestimation of the number of colonies. This is better shown in Figure 7b, where it is pointed out that the error in the estimated number of colonies (i.e., absolute value of the difference between the number of colonies measured with the proposed system and the number obtained with PCT) is negligible when the sample microbial concentration is lower than 1200 CFU/mL (i.e., less than 24 colonies are present). In Figure 7c, the scatter plot of the number of detected colonies vs. the sample microbial concentration is shown for the subset of investigated samples featuring a microbial concentration lower than 1200 CFU/mL. As can be seen, in this case, the number of detected colonies is a linear function of the sample microbial concentration and the value of the determination coefficient (R^2^ = 0.9735) is high.

### 3.3. Towards an Embedded Electronic System

The presented computer vision sensor system has been in-house validated using benchtop instrumentation in a laboratory environment. However, it can also be implemented in the form of an embedded electronic instrument using low-cost electronics to be used outside a laboratory by non-trained personnel. This can be very useful, for example, in the case of small production environment that cannot afford an internal laboratory for quality testing, to avoid the costs and long response delay due to sample shipping to an external laboratory.

**Figure 8:**
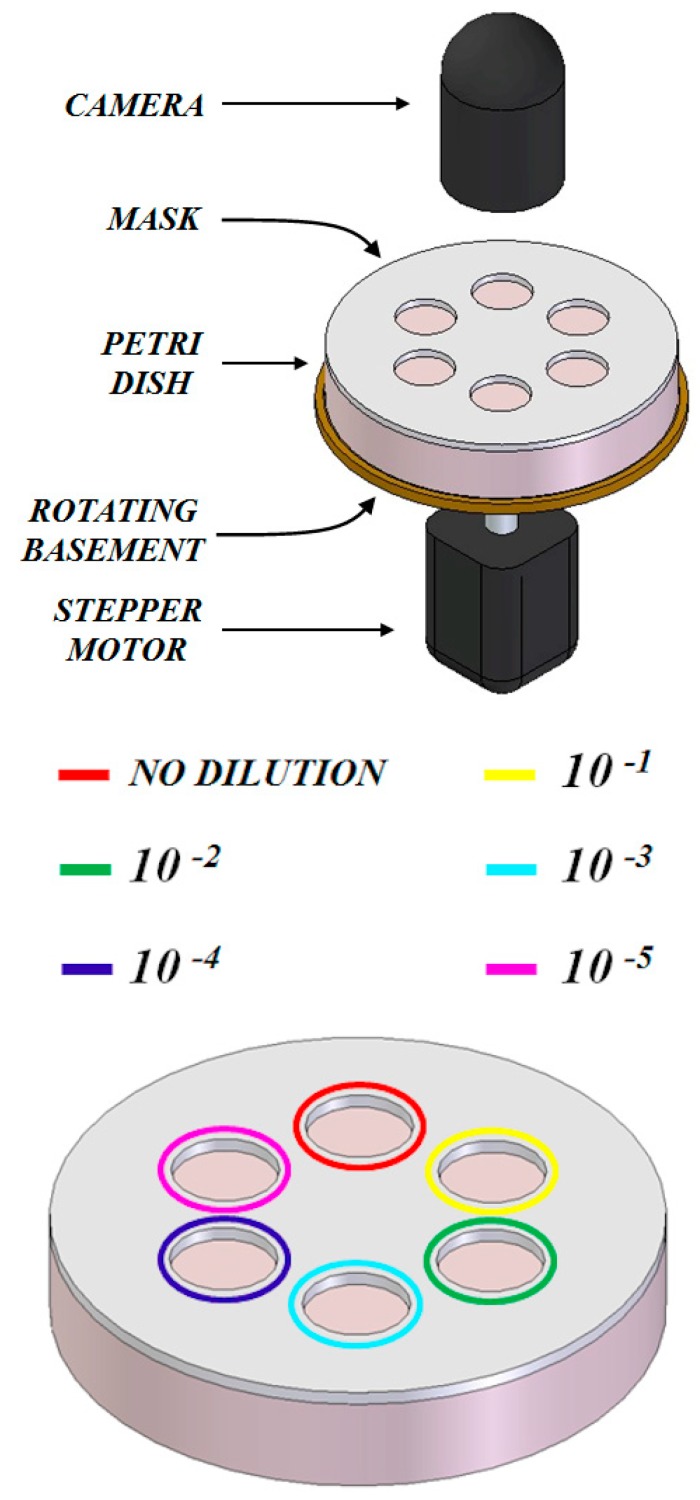
Schematic of a possible implementation of the computer vision sensor system proposed in this work in the form of an embedded electronic instrument for microbial analysis outside a laboratory environment.

The schematic of the implementation of the computer vision sensor system in the form of an embedded electronic system is presented in Figure 8. The Petri dish features six different active areas, where 20 µL of six different dilutions (no dilution, 10^−1^, 10^−2^, 10^−3^, 10^−4^, 10^−5^) of the sample are inoculated. The Petri dish is put on a rotating basement operated by a stepper motor that can place each of the six active areas under the camera to take a picture. All the system is located in a thermoregulated chamber where the target temperature to favour microbial growth is set.

At the beginning of the assay the Petri dish is placed on the rotating platform inside the thermal chamber set at the target temperature. Every ten minutes a picture of each active area on the Petri dish is taken and saved on a non-volatile memory. After the end of the assay, the pictures are processed as discussed in Section 2. The number of colonies and growth kinetics parameters are then determined by choosing the active area featuring a number of detected colonies lower than 20 and compensating for the corresponding dilution factor.

All the system can be built using low-cost electronics and controlled by a microcontroller in charge of data acquisition from the camera, data saving to a flash memory embedded in the developed electronic board, control of the chamber temperature and data processing. The thermoregulation system can be implemented, for example, using a Peltier cell, capable of both heating and cooling, controlled using a proportional-integral-derivative (PID) algorithm implemented on the microcontroller.

## 4. Conclusions

In this paper, a computer vision sensor system for microbial concentration detection has been presented. The system can estimate the microbial concentration of the sample under test by continuously monitoring a Petri dish and detecting the colonies grown on the dish using a blob detection algorithm. The system is also capable of measuring the lag time and growth rate of the detected colonies, thus also providing information on the microbial contaminant kinetics growth parameters.

All the investigations have been carried out in a laboratory environment using benchtop instrumentation. However, such a system can be implemented in the form of an embedded electronic system built with low-cost electronics to perform microbial contamination analysis also outside a laboratory by non-trained personnel. This can be of great importance in particular for small sized production centers that cannot afford the cost of an internal laboratory. The system can provide the results in a short time, thus avoiding the long delays related to shipping the samples to an external laboratory for analysis.

## Figures and Tables

**Table 1:** Growth kinetics parameters for the different microbial species and growth media investigated in this work. The parameters b and c are defined as in Equation (1). Average value and standard deviation for the parameters are provided.

*Kinetics* *Parameter*	*E. coli* (NA)	*E. coli* (BHI)	*S. aureus* (NA)	*S. aureus* (BHI)	*P. aeruginosa* (NA)	*S. cerevisiae* (SDA)
b	572.65 ± 1.63	473.77 ± 7.64	1025.1 ± 153.2	1045.1 ± 69.93	727.9 ± 13.71	1502.9 ± 110.1
c	(6.91 ± 1.21)∙10^−4^	(1.11 ± 0.1)∙10^−3^	(2.13 ± 0.04)∙10^−4^	(4.85 ± 1.15)∙10^−4^	(9.01 ± 0.56)∙10^−4^	(2.86 ± 0.44)∙10^−4^

## References

[1] Justino C.I., Duarte A.C., Rocha-Santos T.A. (2017). Recent progress in biosensors for environmental monitoring: A review. Sensors.

[2] Partyka M.L., Bond R.F., Chase J.A., Atwill E.R. (2017). Monitoring bacterial indicators of water quality in a tidally influenced delta: A Sisyphean pursuit. Sci. Total Environ..

[3] Välimaa A.L., Tilsala-Timisjärvi A., Virtanen E. (2015). Rapid detection and identification methods for Listeria monocytogenes in the food chain—A review. Food Control.

[4] Mizan M.F.R., Jahid I.K., Ha S.D. (2015). Microbial biofilms in seafood: A food-hygiene challenge. Food Microbiol..

[5] Bertelli C., Greub G. (2013). Rapid bacterial genome sequencing: Methods and applications in clinical microbiology. Clin. Microbiol. Infect..

[6] Fournier P.E., Drancourt M., Colson P., Rolain J.M., La Scola B., Raoult D. (2013). Modern clinical microbiology: New challenges and solutions. Nat. Rev. Microbiol..

[7] Scallan E., Hoekstra R.M., Angulo F.J., Tauxe R.V., Widdowson M.A., Roy S.L., Jones J.L., Griffin P.M. (2011). Foodborne illness acquired in the United States—Major pathogens. Emerg. Infect. Dis..

[8] Reasoner D.J. (2004). Heterotrophic plate count methodology in the United States. Int. J. Food Microbiol..

[9] Ladero V., Martínez N., Martín M.C., Fernández M., Alvarez M.A. (2010). qPCR for quantitative detection of tyramine-producing bacteria in dairy products. Food Res. Int..

[10] Sedgley C., Nagel A., Dahlén G., Reit C., Molander A. (2006). Real-time quantitative polymerase chain reaction and culture analyses of Enterococcus faecalis in root canals. J. Endod..

[11] Carbonnelle E., Mesquita C., Bille E., Day N., Dauphin B., Beretti J.L., Ferrini A., Gutmann L., Nassif X. (2011). MALDI-TOF mass spectrometry tools for bacterial identification in clinical microbiology laboratory. Clin. Biochem..

[12] Clark A.E., Kaleta E.J., Arora A., Wolk D.M. (2013). Matrix-assisted laser desorption ionization–time of flight mass spectrometry: A fundamental shift in the routine practice of clinical microbiology. Clin. Microbiol. Rev..

[13] Boehm D.A., Gottlieb P.A., Hua S.Z. (2007). On-chip microfluidic biosensor for bacterial detection and identification. Sens. Actuators B Chem..

[14] Singh A., Poshtiban S., Evoy S. (2013). Recent advances in bacteriophage based biosensors for food-borne pathogen detection. Sensors.

[15] Wang Y., Ye Z., Ying Y. (2012). New trends in impedimetric biosensors for the detection of foodborne pathogenic bacteria. Sensors.

[16] Ercole C., Del Gallo M., Mosiello L., Baccella S., Lepidi A. (2003). Escherichia coli detection in vegetable food by a potentiometric biosensor. Sens. Actuators B Chem..

[17] Nik Mansor N., Leong T., Safitri E., Futra D., Ahmad N., Nasuruddin D., Itnin A., Zaini I., Arifin K.T., Heng L.Y. (2018). An amperometric biosensor for the determination of bacterial sepsis biomarker, secretory phospholipase group 2-IIA using a tri-enzyme system. Sensors.

[18] Grossi M., Riccò B. (2017). Electrical impedance spectroscopy (EIS) for biological analysis and food characterization: A review. J. Sens. Sens. Syst..

[19] Kleiss T., Albrecht J., Putallaz T., Cordier J.L. (1995). Impedance measurement of the microbial flora in dairy-based desserts. J. Microbiol. Methods.

[20] Grossi M., Lanzoni M., Pompei A., Lazzarini R., Matteuzzi D., Ricco B. A portable biosensor system for bacterial concentration measurements in cow’s raw milk. Proceedings of the 4th IEEE International Workshop on Advances in Sensors and Interfaces (IWASI).

[21] Hardy D., Kraeger S.J., Dufour S.W., Cady P. (1977). Rapid detection of microbial contamination in frozen vegetables by automated impedance measurements. Appl. Environ. Microbiol..

[22] Bülte M., Reuter G. (1984). Impedance measurement as a rapid method for the determination of the microbial contamination of meat surfaces, testing two different instruments. Int. J. Food Microbiol..

[23] Grossi M., Lazzarini R., Lanzoni M., Pompei A., Matteuzzi D., Riccò B. (2013). A portable sensor with disposable electrodes for water bacterial quality assessment. IEEE Sens. J..

[24] Grossi M., Parolin C., Vitali B., Riccò B. (2018). A portable sensor system for bacterial concentration monitoring in metalworking fluids. J. Sens. Sens. Syst..

[25] Zhou X., King V.M. (1995). An impedimetric method for rapid screening of cosmetic preservatives. J. Ind. Microbiol..

[26] Gomez-Sjoberg R., Morisette D.T., Bashir R. (2005). Impedance microbiology-on-a-chip: Microfluidic bioprocessor for rapid detection of bacterial metabolism. J. Microelectromech. Syst..

[27] Grossi M., Parolin C., Vitali B., Riccò B. (2019). Measurement of bacterial concentration using a portable sensor system with a combined electrical-optical approach. IEEE Sens. J..

[28] Ates H., Gerek O.N. An image-processing based automated bacteria colony counter. Proceedings of the 24th International Symposium on Computer and Information Sciences.

[29] Brugger S.D., Baumberger C., Jost M., Jenni W., Brugger U., Mühlemann K. (2012). Automated counting of bacterial colony forming units on agar plates. PLoS ONE.

[30] Commercial Colony Counter. http://iul-instruments.com/product/sphereflash-automatic-colony-counter/.

[31] Griffith R.V., McMahon T.A., Espinosa G. (1984). A commercial bacterial colony counter for semiautomatic track counting. Nucl. Tracks Radiat. Meas..

[32] Grossi M. (2019). A sensor-centric survey on the development of smartphone measurement and sensing systems. Measurement.

[33] Minoi J.L., Chiang T.T., Lim T., Yusoff Z., Karim A.H.A., Zulharnain A. Mobile vision-based automatic counting of bacteria colonies. Proceedings of the International Conference on Information and Communication Technology (ICICTM).

[34] Poladia M., Fakatkar P., Hatture S., Rathod S.S., Kuruwa S. Detection and analysis of waterborne bacterial colonies using image processing and smartphones. Proceedings of the International Conference on Smart Technologies and Management for Computing, Communication, Controls, Energy and Materials (ICSTM).

[35] Blob Detection Algorithm, Principle of Work and Code in JAVA. http://www.labbookpages.co.uk/software/imgProc/blobDetection.html.

[36] Yuen H.K., Princen J., Illingworth J., Kittler J. (1990). Comparative study of Hough transform methods for circle finding. Image Vis. Comput..

